# Supplementary material for: Discrimination of Natural Mature Acacia Honey Based on Multi-Physicochemical Parameters Combined with Chemometric Analysis
Source: Molecules. 2019 Jul 23;24(14):2674. doi: 10.3390/molecules24142674 (PMC6680568; doi:10.3390/molecules24142674)
Supplement: Supplementary file 1 [file molecules-24-02674-s001.pdf]

Physicochemical characteristics

The methods used for the quantitative analysis of physicochemical properties were determined according to the Association of Official Analytical Chemists (AOAC 1990). AOAC Official Method 969.38 Moisture; AOAC Official Method 960.44 Color; AOAC Official Method 957.16 Conductivity; AOAC Official Method 994.16 pH; AOAC Official Method 962.19 Acidity (Free, Lactone, and Total); AOAC Official Method 954.11 Sugar; AOAC Official Method AOAC Official Method 2001.11 protein; AOAC Official Method 958.09 amylase activity; AOAC Official Method 979.20 proline; AOAC Official Method 980.23 Hydroxymethylfurfural;

Total Phenolic Content (TPC) was measured using Folin–Ciocalteu reagent (Singleton, Orthofer, & Rosa，1999). Honey samples were prepared at a concentration of 100 mg/mL and 1mL of filtrate was mixed with 1 mL of Folin–Ciocalteu reagent solution and then 5 mL of sodium carbonate solution was added (1mol/L) and mixed. After one hour of light proof reaction at room temperature, the absorbance of samples was measured at a wavelength of 760 nm by the simultaneous spectrophotometric. A standard curve was plotted using gallic acid in the range from 10 to 200 mg/L (R^2 = 0.9987) and results were expressed as mg of gallic acid equivalent (GAE) per 1000 g of honey sample (mg GAE/kg).

Determination of glucose oxidase in honey refers to the method of Zhou Jianqin et al. (2008) with some modifications. Honey sample (10g) dissolved in distilled water (100 mL), as honey test solution. After heating 30 min in 37 °C water bath pot, 1 mL test samples was pipetted into a 10 mL brown test tube, and 1.2 mL of acetic acid-sodium acetate buffer (pH 3.5) and 520 μL of indigo carmine red dye solution were added, diluted to calibration line with distilled water and shook evenly. Heated in 100 °C water bath pot for 13 min, and immediately placed under running water for 5 min. After using distilled water as a reference, the absorbance of samples was measured at a wavelength of 615 nm by the simultaneous spectrophotometric. The amount of hydrogen peroxide (μg/g) produced by glucose oxidase oxidation of glucose in honey within 30 min was calculated by means of a hydrogen peroxide standard curve, which is its

glucose oxidase activity.

Singleton, V. L., Orthofer, R., & Rosa M Lamuela-Raventós. (1999). Analysis of total phenols and other oxidation substrates and antioxidants by means of folin-ciocalteu reagent. *Methods in enzymology*, *299C(1)*, 152-178. http:// 10.1016/S0076-6879(99)99017-1

Zhou, J. Q., Chen, S. H., & Wang, J. W. (2008). A simple and convenient method to determine the activity of glucose oxidase. *Experimental Technology & Management*, 25(12), 58-60. http:// 10.3969/j.issn.1002-4956.2008.12.016

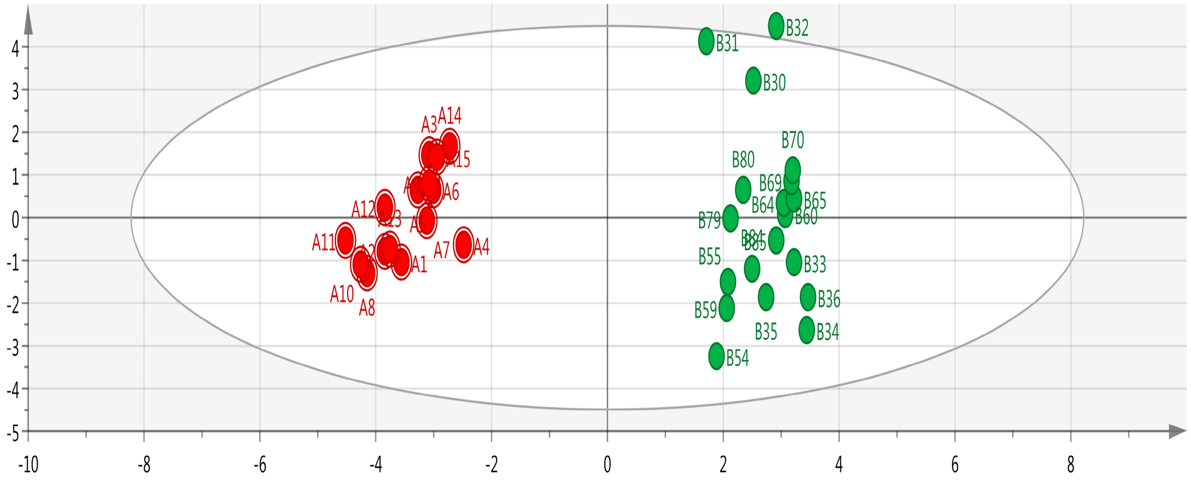

Supplementary Figure S1. PCA score plots

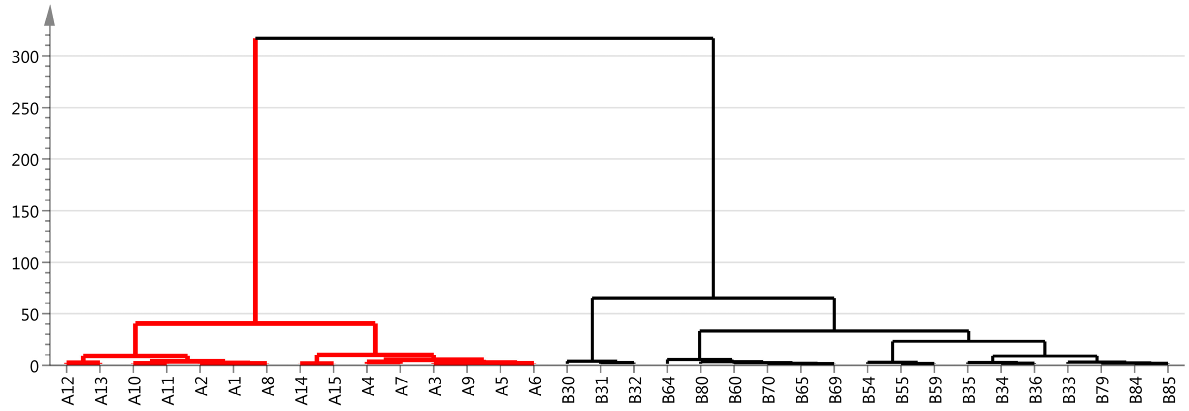

Supplementary Figure S2. Results of hierarchical cluster analysis of test sample

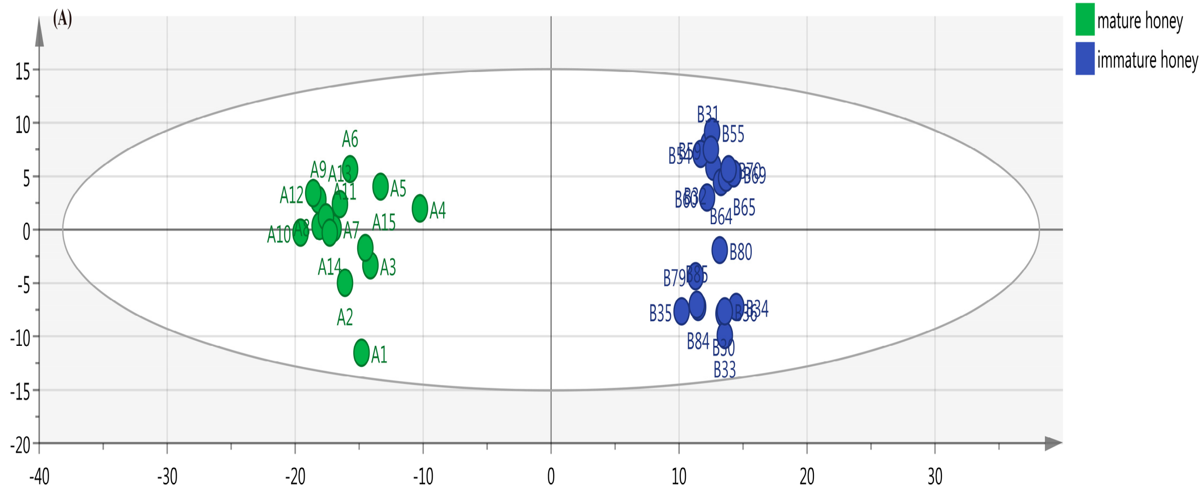

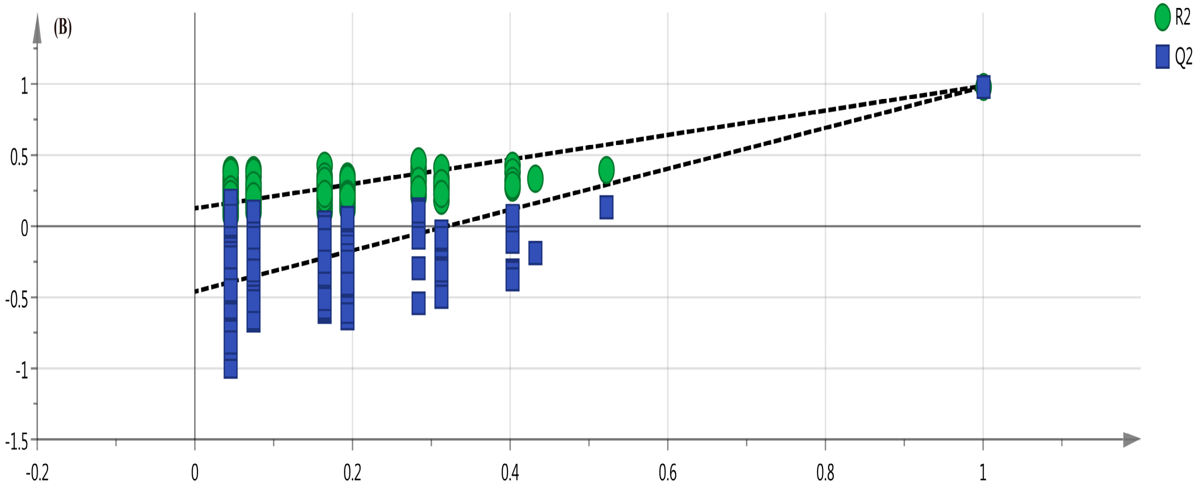

Supplementary Figure S3. The OPLS-DA score plots (A) and OPLS-DA validation plot (B)
